# Properties of metabolic graphs: biological organization or representation artifacts?

**DOI:** 10.1186/1471-2105-12-132

**Published:** 2011-05-04

**Authors:** Wanding Zhou, Luay Nakhleh

**Affiliations:** 1 Department of Bioengineering, Rice University, Houston, Texas, USA; 2 Department of Computer Science, Rice University, Houston, Texas, USA

## Abstract

**Background:**

Standard graphs, where each edge links two nodes, have been extensively used to represent the connectivity of metabolic networks. It is based on this representation that properties of metabolic networks, such as hierarchical and small-world structures, have been elucidated and null models have been proposed to derive biological organization hypotheses. However, these graphs provide a simplistic model of a metabolic network's connectivity map, since metabolic reactions often involve more than two reactants. In other words, this map is better represented as a hypergraph. Consequently, a question that naturally arises in this context is whether these properties truly reflect biological organization or are merely an artifact of the representation.

**Results:**

In this paper, we address this question by reanalyzing topological properties of the metabolic network of *Escherichia coli *under a hypergraph representation, as well as standard graph abstractions. We find that when clustering is properly defined for hypergraphs and subsequently used to analyze metabolic networks, the scaling of clustering, and thus the hierarchical structure hypothesis in metabolic networks, become unsupported. Moreover, we find that incorporating the distribution of reaction sizes into the null model further weakens the support for the scaling patterns.

**Conclusions:**

These results combined suggest that the reported scaling of the clustering coefficients in the metabolic graphs and its specific power coefficient may be an artifact of the graph representation, and may not be supported when biochemical reactions are atomically treated as hyperedges. This study highlights the implications of the way a biological system is represented and the null model employed on the elucidated properties, along with their support, of the system.

## Background

Graphs have been used extensively to model the connectivity of cellular processes [1], including metabolic networks [2]. Once represented as a graph, a wide array of tools can be applied to visualize and analyze the graph to elucidate properties of the corresponding cellular network [2,3]. Analyses of metabolic networks based on the graph representation have revealed a wide range of significant properties of the network connectivity, including a short mean path length [4], a scale-free degree distribution [5] and a bow-tie structure [6]. The statistical significance of such findings, and whether these graph features have been subject to adaptive evolution, are often assessed by comparing biological networks to networks generated under null models. In this context, null models produce random (standard) graphs that are constrained to satisfy one or more requirements, such as an expected degree distribution. However, in metabolic networks, a reaction often involves more than two reactants, rendering standard graphs too simplistic and consequently requiring a certain abstraction. For example, one commonly used techniques for enabling a graph representation of a metabolic network's connectivity map is to model each reaction by a complete subgraph, where each pair of reactants on both sides of the reaction are linked by an edge. Analyses based on different representations of the metabolic network of *E. coli *have revealed conflicting patterns related to its small-worldness [5,7,8]. It is therefore natural to ask whether these properties, that are elucidated based on a standard graph representation and a null model, truly reflect biological organization or are merely an artifact of the representation.

**Figure 1:**
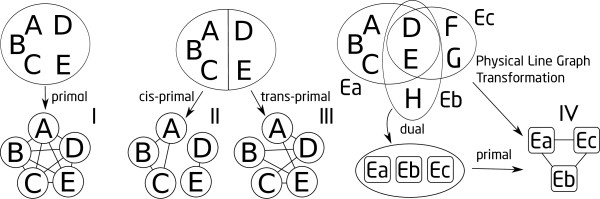
**Illustration of the hypergraph transformations and abstractions**. Left: a hyperedge is turned into a complete graph linking every pair of nodes to obtain the primal graph (I). Middle: the *cis*- (II) and *trans*-primal (III) graphs are obtained by connecting either nodes in the same side of the hyperedge partition or on different sides, respectively. Right: The physical line graph transformation (IV) can be obtained by taking the primal of the dual of the hypergraph; that is, it is the composition of two transformations.

To investigate this question, we analyze metabolic network connectivity maps from a *hypergraph *perspective. Given that metabolic reactions may involve more than two reactants, hypergraphs--where an edge connects any finite number of nodes--provide a more realistic model of the connectivity of a metabolic network. Indeed, Klamt et al. [9] recently argued that any metabolic (standard) graph representation fails to describe the dependence of a metabolite on others that participate in the same reaction. They illustrated that even a bipartite graph, with metabolites and reactions being the two node types, fails to remedy the problem [9] as links in bipartite graphs still remain independent. Further, Lacroix et al. [2] suggested that each reaction has to be taken as a whole (yet did not specify how to analyze such data). To properly represent reactions that involve more than two entities, hypergraphs (see [10,11] for introductory texts on hypergraphs) are the natural representation of metabolic networks' connectivity maps (e.g., see [9]). A generalization of standard graphs, a hypergraph allows any subset of two or more nodes to form an edge, called a *hyperedge*. Further, to distinguish between the metabolites on different sides of a metabolic reaction, and to allow for the designation of the reaction direction, the set of nodes connected by a hyperedge can be bipartitioned into the *head set *and the *tail set*. Standard graph representation of a metabolic network connectivity is in fact a transformation of the underlying hypergraph. The *substance model *(every pair of substances/metabolites participating in the same reaction are connected by an edge), *substrate-substrate model *(every pair of metabolites on the same side of a reaction are connected by an edge), and *substrate-product *model (every pair of metabolites on opposite sides of a reaction are connected by an edge), discussed in [12], correspond to the *primal, cis-primal*, and *trans-primal*, respectively, of the underlying hypergraph. These transformations on hypergraphs are formally defined in the Methods section below, and are illustrated in Figure 1.

Some work on metabolic connectivity hypergraphs already exists. For example, Forst et al. [13] used algebraic operations to compare metabolic hypergraphs across multiple species for phylogenetic reconstruction. A directed hypergraph-based tool, Rahnuma, has been developed recently for metabolic pathway analysis [14]. An algorithm for computing the *minimal cutting set *on hypergraphs was proposed [15].

Further, it is worth pointing out that the hypergraph property of the dependence among metabolites participating in the same reaction has already been widely, though implicitly, captured in other modeling techniques, such as network expansion [16], reachability analysis [17], constraint-based modeling [18] and Petri-net modeling [19]. For example, the stoichiometric matrix used in the constraint-based modeling is essentially a weighted incidence matrix of the underlying hypergraph (where each column corresponds to a hyperedge). This again reflect s the rather natural view that metabolic network connectivity maps are inherently hypergraph-like. Nonetheless, with the exception of these very few studies, most analyses of metabolic networks' connectivity maps in the literature are based on (standard) graph representations. This lack of adoption of hypergraphs may owe to a host of factors. One of them is the inherent difficulty in visualization [20]. Obtaining an informative hypergraph layout is much more involved than obtaining a standard graph layout (see [21] for a typical algorithm for drawing hypergraphs under the *subset standard*). Besides, many problems that can be solved efficiently on standard graphs become NP-hard on hypergraphs (e.g., the problem of finding the shortest-path in a hypergraph with hyperedges weighted by their cardinalities [22]). Finally, the lack of well-defined hypergraph counterparts to the common standard graph characteristics, such as clustering coefficients, may have made their use less appealing.

In this paper, we address the aforementioned question by conducting three tasks on the metabolic network connectivity map of *Escherichia coli*. First, we analyze the scaling of degree distributions [4,7] and average local clustering coefficients [23] on various standard graph abstractions. While a host of topological properties can be analyzed, we focus on these properties since they are central to the two aforementioned hypotheses about metabolic networks. Then, we show how these analyses are affected when the null model incorporates the reaction size (hyperedge cardinality)--a quantity that, to the best of our knowledge, is ignored in existing studies. Finally, we devise measures of local and global clustering coefficients that apply directly to hypergraphs and differ from those of Estrada and Rodríguez-Velázquez [24] in their satisfaction of desired properties. Based on these three tasks we find that a null model that incorporates the hyperedge cardinalities changes the analysis results significantly compared to the previously used null models.

Further, when clustering is analyzed directly on the hypergraph representation, the scaling property, which has been reported in the literature, becomes poorly supported. These results combined suggest that the reported scaling of the clustering coefficients in the metabolic graphs and its specific power coefficient may be an artifact of the graph structure produced by the abstraction process and may not be supported when biochemical reactions are atomically treated as hyperedges. This study highlights the implications of the systems representation and null model employed in an analysis on the hypotheses derived for that system. Further, these results have implications beyond metabolic networks since, for example, signal transduction networks contain many enzymatic and complexing reactions that form hyperedges. The weakening of statistical support of reported properties of biological networks when the new null model is considered calls into question claims that adaptive evolution is the (only) explanation for the emergence of complex, or non-intuitive, network features. More generally, this study further emphasizes the issue that the use of proper representations and null models is fundamental to understanding the biology underlying the abstract model.

## Results and Discussion

### A Binomial Distribution of Reaction Sizes and Its Effects

When transforming a hypergraph into a standard graph, under any of the aforementioned transformations, the information on the hyperedge cardinality is lost. The question, then, is whether ignoring the hyperedge cardinality distribution affects the properties elucidated from abstracted standard graphs. Further, if the answer is positive, how should this information be integrated into null models of generating random metabolic graphs in analytical studies.

To address the first question, we begin by inspecting the degree distributions of primal graphs generated randomly in a way to account for hyperedge constraints. It is analytically very hard to establish the degree distribution of the primal of randomly generated hypergraphs, since the overlap between hyperedges creates dependencies among the degrees of the nodes. Therefore, we study this issue in simulations. Given a metabolic hypergraph *H *= (*V*, ℰ), where |ℰ| = *m *and the maximum cardinality of any hyperedge *E *∈ ℰ is *k*, the primal of *H *has *ℓ *edges, where *ℓ *≤ *m *· *k*(*k *- 1)/2. One method for generating random (standard) graphs in this context, while accounting for a fixed hyperedge cardinality *k *is to use *m *as the constraint; i.e., generate a hypergraph with *m *hyperedges, each of cardinality *k*, and compute its primal. In other words, a hyperedge of cardinality *k *is generated by randomly sampling (without repeats) a subset of *k *nodes and connecting them by a hyperedge, and the process is repeated *m *times (another method is to generate "enough" hyperedges, each of cardinality *k*, in the hypergraph to yield (approximately) *ℓ *edges in its primal; see additional file 1).

**Figure 2:**
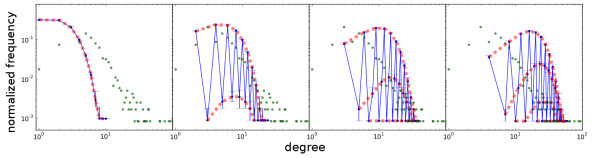
**The degree distributions of the primal graphs of random hypergraphs**. Each of the hypergraphs has 1193 nodes and 1168 hyperedges. Columns from left to right correspond to fixed hyperedge cardinalities of 2, 3, 4, and 5, respectively. The results in each panel are based on the 300 randomly generated hypergraphs (replica). For each well represented degree value (contained in at least 10 replica), the median is plotted. Error bars indicate quartiles. Green dots correspond to the degree distribution of the primal graph of the (undirected) metabolic hypergraph of *E. coli*. All plots are on log-log scales.

In the case of the *E. coli *metabolic network, the hypergraph has *n *= 1193 nodes and *m *= 1168 hyperedges, and its primal has *ℓ *= 5718 edges. For each combination of *n*, *m*, *ℓ *and hyperedge cardinality *k *∈ {2, 3, 4, 5}, we generated 300 random (standard) graphs based on the above method, and plotted the median degree distributions of these graphs, along with that of the primal of the metabolic hypergraph of *E. coli*. The results are shown in Figure 2, where the four panels, from left to right, correspond to fixed hyperedge cardinalities of *k *= 2, 3, 4, 5, respectively (see additional file 1 for results based on the other random graph generation method, as well as the relationship between the two).

Notice that hypergraphs with different hyperedge cardinalities give rise to standard graphs with different degree distributions. In general, the degree distribution of the primal of a random undirected hypergraph with hyperedge cardinality larger than 2 has a zig-zag shape when the degree value is low and becomes more complex as the degree value increases. This is due to the fact that the metabolic hypergraphs we consider are very sparse.

In a hypergraph with *n *nodes, the maximum number of distinct hyperedges of cardinality *k*, for 2 ≤ *k *≤ *n*, is . And, if we exclude the trivial hyperedges (those that have a single node or the entire set of nodes), the maximum number of distinct hyperedges is

In the case of the *E. coli *metabolic network, we have 1168 hyperedges on a set of 1193 nodes. Even if we consider only standard edges (hyperedges of cardinality 2), this hypergraph is very sparse, since the maximum number of distinct hyperedges of cardinality 2 is 1193 * 1192/2 = 711028 which is ≫ 1168. Now, consider a node *v *that is included in only two hyperedges each of which is of cardinality *k*. If the hypergraph is sparse, the probability that the two hyperedges would share nodes besides *v *is very low. Therefore, the primal of this hypergraph is more likely to have node *v *with degree 2*k *- 2 than with degree in between *k *to 2*k *- 3. In other words, since each hyperedge contributes *k *- 1 to the degree of each of its nodes in the primal, more nodes with degrees at integer folds of *k *- 1 are observed if the underlying hypergraph is sparse (when contributions from different hyperedges have less chance to overlap). Hence, it might be visually desirable to classify the degree values into *k *- 1 equivalence classes by *d*_1 _≡ *d*_2 _(mod *k *- 1) ("mod" denotes the modulo operation) and connect data inside each equivalence class (dashed bold lines in Figure 2).

Clearly, the hypergraphs of different hyperedge cardinalities contribute to different but overlapping ranges of degree values. In particular, the leftmost panel of Figure 2 corresponds to the binomial degree distribution of random Erdös-Rényi graphs [25] with 1168 edges and probability *p *= 1168/711028 ≈ 0.001 of linking two randomly chosen nodes by an edge. The degree distribution of the primal of metabolic hypergraphs is a mixture of degree distribution obtained based on different hyperedge cardinalities.

**Figure 3:**
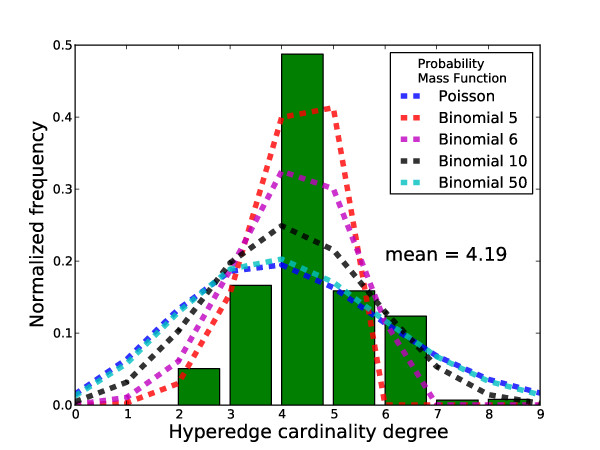
**The hyperedge cardinality distribution of the metabolic hypergraph of *E. coli***. Poisson distribution and Binomial distribution with different sample sizes are shown in dashed lines. Parameters of these distributions (*μ *for Poisson and *p *for binomial) are chosen such that their means equal the actual value (419).

Indeed, in the case of metabolic hypergraphs, neither do all the hyperedge cardinalities take one same value nor do they follow a simple uniform distribution. Their effect on the properties of the abstracted standard graphs has not been studied. In Figure 3 we plot the hyperedge cardinality distribution of the *E. coli *metabolic hypergraph. The mean value of the distribution is 4.19 and the range is roughly from 2 to 10. A comparison to Poisson and binomial distributions show that the shape is narrower than a Poisson distribution with the same mean and is much closer to a binomial distribution with sample size of 5 (see additional file 1 for similar results obtained for other organisms).

### Incorporating the Reaction Size Distribution Into a Null Model

**Figure 4:**
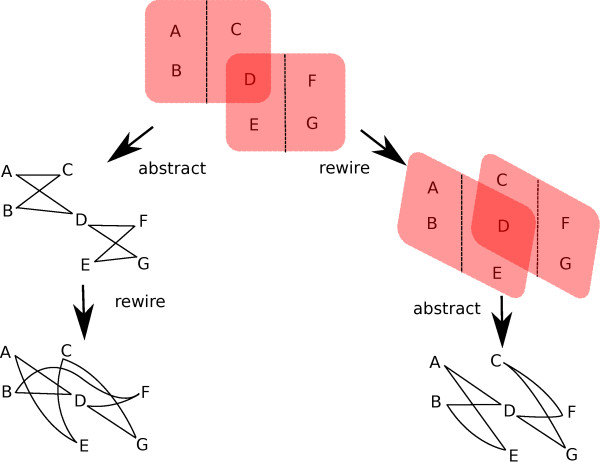
**Comparison of the two null models on a toy hypergraph**. The hypergraph-graph abstraction follows the *trans*-primal procedure. Left: Traditional way of the null model generation by first abstracting the hypergraph and then rewiring. Right: New way of null model generation, maintaining the reaction size distribution, by first rewiring the hypergraph and then abstracting it. Note that the rewiring process does not necessarily preserve the overlapping reactants (e.g., reactant D in the toy hypergraph).

Based on the above results, we believe it is important for a null model for generating random graphs in the context of metabolic networks to use both the number and cardinality distribution of hyperedges. We study a null model where a random graph is generated from the metabolic hypergraph by first rewiring the hypergraph (thus, keeping the number and cardinality distribution of hyperedges unchanged) and then abstracting the random hypergraph (through a *trans*-primal transformation) into a standard graph. We compare the degree distribution of the real metabolic graph against the new null model and another null model that rewires the metabolic standard graph (also through a *trans*-primal transformation from the metabolic hypergraph) directly (see Figure 4 for an illustration of the generation of the null models on a toy hypergraph). Notice that this wiring process does not guarantee that the generated random networks are mass balanced; this is a very important constraint, but integrating it into a random network generation procedure is beyond the scope of this paper.

To rewire the metabolic standard graph of *E. coli*, we perform 20,000 operations each of which randomly removes an edge and links a new pair of previously unconnected nodes. Similarly, to rewire the metabolic hypergraph of *E. coli*, we perform 20,000 operations each of which randomly removes a hyperedge, resamples a new set of nodes of the same size (same size for the tail set and the head set if a directed hypergraph is concerned), and connects the new set with a hyperedge. In this way, we keep the number and cardinality distribution of hyperedges unchanged along the rewiring process. Further, we make sure that the same set of nodes is not selected more than once, to keep all hyperedges distinct. Finally, to obtain statistically significant results, we generate 200 random networks, each of which is rewired in both ways as above 20,000 times.

**Figure 5:**
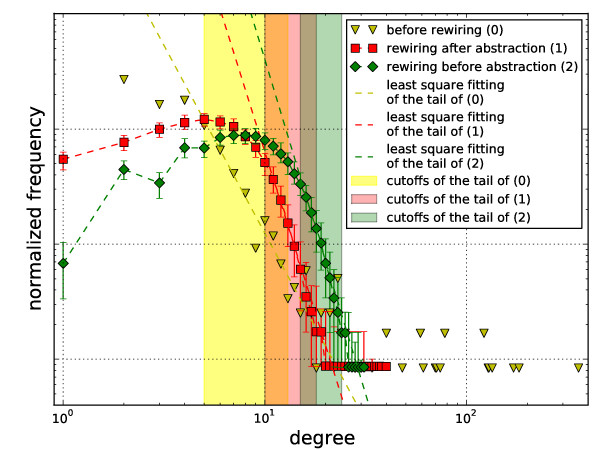
**Comparison of the degree distributions of the metabolic standard graph of *E. coli *against two different null models**. The degree distributions are derived based on three versions of the metabolic hypergraph of *E. coli: *The *trans*-primal of the hypergraph (0), the rewired *trans*-primal of the hypergraph (1), and the *trans*-primal of the rewired hypergraph (2). Least-squares fitting of the tail of (0) and the medians of (1) and (2) to *p*(*k*) = *βk*^-*α *^yields values of *α *= 3.26 for (0), *α *= 5.59 for (1), and *α *= 5.82 for (2). For degree 10^0 ^= 1, the point for (0) coincides with that for (1).

The degree distributions of the *trans*-primal of *E. coli*'s metabolic network and the random networks generated by the two rewiring procedures are shown in Figure 5. Each data point and its error-bar indicate the median, 5-th and 95-th percentiles, respectively. Since not all the degrees are well represented in all 200 replicas, we plot results only for degree values present in at least 10 replicas.

We also fit the tail of the degree distribution of the standard graph of *E. coli *and the median of the rewired graphs to *p*(*k*) = *βk*^-*α *^using the least squares fitting. By inspecting the data, the fitting region for standard graphs is manually set to [5,13] (shaded region in Figure 5). For rewired graphs, the end of the fitting region is defined as the smallest degree at which the 95-th percentile is higher than the frequency at count 1 (in other words, 95% of the replicas have more than one nodes with this degree). The start of the fitting region is determined by finding the first pair of neighboring degrees with slope in medians below a certain threshold (4.0) as one moves from the end of the fitting region to degree 1. We set our fitting region as such since (1) existing studies have focused on fitting degree distributions excluding their heads, for detecting "scale-freeness" [26], and (2) real-world degree distributions are always constrained by the fact that the frequency has to be no smaller than the one corresponding to count 1 (since 0 is invalid on a log-log plot).

Two observations are in order based on Figure 5:

1. The tail shifts to the higher degree region in the graphs abstracted after rewiring the metabolic hypergraph compared with the graphs rewired after being abstracted from the real metabolic hypergraph. Comparison with similar situation in undirected hypergraphs (Figure 2) indicates contribution from higher-order hyperedge cardinality.

2. The *trans*-primal of the rewired hypergraph preserves the zig-zag pattern in the low-degree region of the distribution (the head). The rewired *trans*-primal graphs, on the other hand, lose such shape in its "head". This indicates that the zig-zag pattern in the low degree region of the original degree distribution is due to abstracting the hypergraph with a certain hyperedge cardinality distribution into a standard graph.

These two observations are in agreement with the statement of Wagner and Fell [5] that "*k*-regular random graphs would be particularly poor statistical models of metabolic networks." However, our observations challenge the use of such a random model for a statistical definition of 'key metabolites'. In particular, the *trans*-primal graphs of repeatedly rewired hypergraphs have a degree distribution whose tail is power-law (just like metabolic networks) and whose head is a zig-zag shape (again, just like metabolic networks). This raises the possibility that while adaptive forces may have shaped the cellular metabolism, neutral evolution forces (mutation, recombination, and random genetic drift) may have defined a large part of the network connectivity. This is in agreement with the observations of Lynch [27] and Wagner [28].

### The scaling of clustering coefficient

It has been proposed that metabolic graphs are *hierarchical *(e.g., [29]), which can be characterized by the scaling of the average clustering coefficient *C*(*k*) of nodes with certain degree *k*, against *k*. For example, Ravasz *et al*. found that *C*(*k*) ∝ *k *^-1 ^for a variety of metabolic networks, including that of *E. coli *[29]. Further, they hypothesized that such a hierarchical structure corresponds to functional organization of the metabolic system. The question we investigate is whether the scaling of clustering of the average clustering coefficient is statistically supported when using a null model that incorporates the reaction size (hyperedge cardinality) distribution.

**Figure 6:**
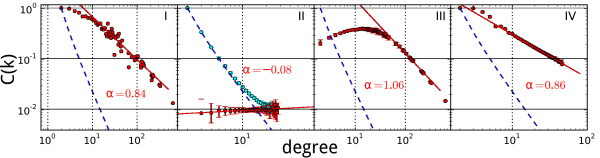
**Scaling of average clustering coefficients *C(k*)**. (I) The primal of *E. coli*'s metabolic hypergraph. (II) Erdös-Rényi random graphs. The cyan dots are *C*(*k*) calculated excluding nodes with *C *= 0. (III) Random graphs with same degree distribution as (I); (IV) primal of rewired versions of *E. coli*'s metabolic hypergraph (see text for more details). For II, III, and IV, the results are based on 100 replica, where the red dots denote the medians of the 100 replica. The red lines are least-squares fitting to *C*(*k*) against *k *using power law. Power coefficients of the fitting are labeled. In each panel, the dashed blue curve corresponds to the points [*k*, γ*_k_*], where γ*_k _*is the smallest *C *value that a node with degree *k *can take.

In Figure 6, we show average clustering coefficient as a function of node degrees, *C*(*k*), for four types of graphs:

(I) The primal of the *E. coli *hypergraph (1193 nodes and 5719 edges).

(II) Erdös-Rényi random graphs with 1193 nodes and 5719 randomly chosen edges.

(III) Random graphs generated by 100,000 rewiring operations applied to the graph in (I), where in each rewiring operation, a pair of non-adjacent edges are selected, and the neighbors of an endpoint of one edge are swapped with the neighbors of an endpoint of the other edge. This procedure generates random graphs with the same degree distribution as that of the graph in (I).

(IV) The primal of hypergraphs generated by 100,000 rewiring operations applied to the *E. coli *metabolic hypergraph (the same method used in the previous section).

Very similar patterns were observed when taking *cis*-primals of directed hypergraphs. Slight difference in *trans*-primals of directed hypergraphs is due to the break of the clique structure in randomization (see notes in additional file 1).

For an Erdös-Rényi random graph with 1193 nodes and 5710 edges, a small value of *C*(*k*) is expected as the connectivity is very sparse, this is shown in Figure 6(II). However, if we exclude nodes whose clustering coefficient is 0, *C*(*k*) scales almost exactly the same with the smallest non-zero *C *values that a node with a particular degree *k *can take (blue dashed line in Figure 6). This smallest non-zero clustering coefficient equals the reciprocal of the total number of connections among the *k *neighbors of the node we consider, which is 2/(*k*^2 ^- *k*), and thus scales with *α *= 2 when *k *is large (that is, 2/(*k*^2 ^- *k*) ≈ *bk*^-2 ^for large *k*). In other words, for a sparse Erdös-Rényi graph, the scaling of *C *with *α *= 2 is very likely.

If we rewire the primal of *E. coli*'s hypergraph in such a way that we preserve the degree distribution, then we obtain graphs whose tail of clustering coefficient distribution scales with an *α *= 1.06, as shown in Figure 6(III). This, to a certain degree, weakens the statistical significance of the scaling observed in Figure 6(I). However, when we employ the null model like that of the previous section (see Figure 4), where the hyperedge cardinality distribution is preserved, we observe that not only do the clustering coefficients scale, but that the scaling has an almost identical value of *α*; see Figure 6(IV). This finding challenges the statement that hierarchical connectivity of metabolic networks corresponds to functional organization. Or, even if such a correspondence still exists, our finding here does not support the hypothesis that such structure is selected for, since random graphs generated based on the new null model exhibit similar scaling properties.

**Figure 7:**
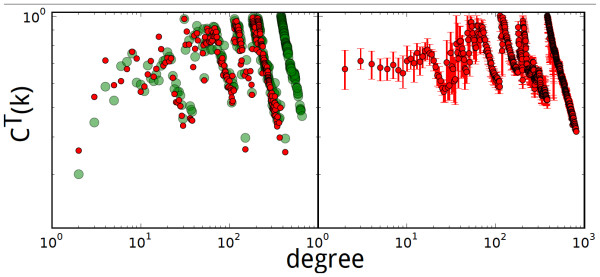
**The scaling of averaged clustering coefficients in the reaction graph obtained via PLGT**. Left panel: The green dots are the average clustering coefficients of the PLGT of *E. coli*'s hypergraph. The red dots are the same, but with the water molecule excluded. Right panel: The red dots are the average clustering coefficients of the rewired hypergraph, which is generated by first taking the dual of *E. coli*'s metabolic hypergraph and then rewiring it (1 × 10^6 ^times, to guarantee convergence).

We also studied the clustering coefficient on reaction graphs obtained through PLGT (see Figure 1). Contrary to the previous observation that the average clustering coefficient *C^T^*(*k*) scales as *C^T^*(*k*) ∝ *k*^0.08 ^[30], *C^T^*(*k*) does not show clear scaling in this study (see Figure 7).

Further, in this case we find that the clustering coefficients are greatly affected by the presence of metabolites that participate in a large number of reactions, or the so-called "currency metabolites", such as water. With water removed from the original hypergraph, the entire rightmost vertical strip in the PLGT's clustering coefficients disappears (red dots in Figure 7) (Effects of the removal of other "currency metabolites" are also studied, see additional file 1). This is because a node with degree *k *becomes a hyperedge with cardinality *k *in the dual hypergraph, giving rise to *k*(*k *- 1)/2 connections in its primal which is the final PLGT product. This has two complications. First, through PLGT, the graph becomes denser. The average degree, or twice the number of edges per node, increased from 9.6 to 228.0. Second, the difference in the contribution to the connection from nodes of different degrees increases significantly, from *k *to *k*(*k *- 1)/2. The node with the largest degree (water) is at least partially responsible for most of the connections in the PLGT result.

The results of *C*(*k*) against k on the PLGT graphs are different from the ones on randomized graphs, whether the graph abstracted is rewired directly or the underlying hypergraph is rewired and abstraction is made thereafter (see additional file 1). However, if the dual hypergraph (of which the PLGT is the primal) is rewired while keeping the number of reactions in which each metabolite participates, the results of *C*(*k*) against *k *on the standard graph abstracted thereafter is similar to the one observed on the PLGT of the *E. coli *hypergraph (right panel of Figure 7). Once again, this result stresses the implications of the used null model, and how this affects the significance of values computed on biological networks.

The question, then, is: why is this scaling of clustering coefficients? Or, why is this hierarchical structure of graphs abstracted from hypergraphs? We believe that this is simply an artifact of the way standard graphs are abstracted from metabolic hypergraphs. For example, the primal of an undirected hypergraph connects all the reactants in the same reaction, thereby forming cliques in the abstracted standard graph. These cliques contribute the same number of 2-paths and triangles in computing the clustering coefficient of a reactant. Since the number and size of such cliques remain unchanged as a hypergraph is rewired, their contribution remains the same as well. The similarity between the scaling of *C*(*k*) in metabolic standard graphs and ones abstracted from randomized hypergraphs indicates that cliques thus formed probably dominate the value of clustering coefficients and thus their scaling in the context of the real-world metabolic networks. In other words, the scaling of *C*(*k*) is kept largely by the hyperedge cardinality distribution which is intrinsic to the structure of biochemical reactions but not to how the metabolic hypergraph is organized using these reactions.

**Figure 8:**
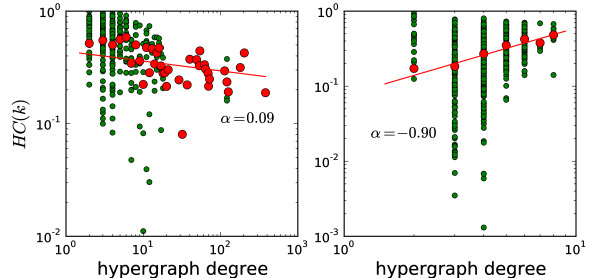
**The scaling of hypergraph clustering coefficient**. The green dots are the local clustering coefficients. The red dots are averaged value of the local clustering ceofficients for each degree. Left panel: *E. coli*'s hypergraph. Right panel: The dual of *E. coli*'s hypergraph.

In order to figure out whether the scaling of clustering coefficients is due to the inherent "hierarchy" of the metabolic graph, or is just a consequence of the graph abstraction process and the hyperedge cardinality distribution, we computed the hypergraph clustering coefficient using a new measure we devised to apply directly to hypergraphs (see Methods). Results are shown in Figure 8 for *E. coli*'s hypergraph (left panel) and its dual (right panel). The result of clustering coefficient computed using the measure of [24] are similar (see additional file 1). The hypergraph average clustering coefficients show very weak scaling. The individual clustering coefficients are more scattered around. The value of *α *(0.09) is much smaller than what is observed on the standard graph (0.84, Figure 6(I)) and the value of 1.1 as reported in [30]. As for the dual hypergraph (right panel of Figure 8), we find that the clustering coefficients of the dual hypergraph, from which the line transformed reaction graph is abstracted, shows better scaling but with an *α *of a larger magnitude. Still, the actual values of the clustering coefficients are very scattered and show no scaling.

To summarize, we believe topological characteristics of metabolic networks, such as scale-free degree distributions and scaling of clustering coefficients, are not necessarily a ground for invoking natural selection or making connections to functional organizations. Instead, these properties may lose statistical significance when a null model taking into account of the reaction sizes is used, and may even disappear when computations are done on the appropriate representation of metabolic networks.

## Conclusion

In this article, we investigated the impact of choosing a null model that incorporates the hypergraph property of the metabolic system such as the reaction size distribution to the networks' connectivity analyses. By reanalyzing the degree distribution and clustering coefficient we found that the reported scaling of the clustering coefficients in the metabolic graphs and its specific power coefficient may be an artifact of the hypergraph abstraction, and is not supported when biochemical reactions are atomically treated as hyperedges. Also we found that by taking into the reaction size distribution, a null model can explain some of the details in the shape of the degree distribution that have not been explained otherwise, further highlighting the necessity of using appropriate null models in exploring adaptive evolution, along with the analysis of their support in biological systems.

## Methods

### Data

We assembled the metabolic hypergraph of *Escherichia coli *using the KEGG database [31]. The presence of a reaction was inferred based on whether there is a gene that is annotated to generate any enzyme that catalyzes the reaction. Reaction formulas, enzyme identities and gene annotations were downloaded from KEGG. We recognize that the metabolic networks thus constructed may not provide a complete coverage of the entire metabolic system in *E*. *coli*. However, this is a common way of constructing metabolic networks in existing studies. Further, since our study is aimed at the differences in properties elucidated from different representations of the same system, a complete coverage, while desirable, is not a necessary prerequisite. The undirected hypergraph representation is obtained by putting all the metabolites in each reaction into a single hyperedge. The directed hypergraph representation is obtained by further separating the metabolites on opposite sides of the reaction into the tail and head sets, respectively. Reaction direction is not considered in this study. Finally, we derived standard graph representations based on transformation operations on hypergraphs that amount to commonly adopted representations in existing studies. In particular, we considered the *substance model*, the *substrate-substrate model *and the *substrate-product model*, which correspond to the *primal*, *cis-primal *and *trans-primal *of a hypergraph, respectively. Further, we considered reaction graphs, where nodes correspond to reactions, and two nodes are connected if their reactions share any reactants; this corresponds to the PLGT of a hypergraph. The hypergraph data and the original reaction lists are available from the author's website: http://www.cs.rice.edu/~wz4/metabolic_hypergraph.tgz.

### Metabolic Hypergraphs

An *undirected hypergraph H *is an ordered pair (*V*, ℰ), where *V *is the set of nodes and ℰ is the set of hyperedges. Each hyperedge *E *∈ ℰ connects, or corresponds to, a subset *V*' ⊆ *V*, where |*V*'| ≥ 2. Hypergraphs are a natural model of the connectivity of metabolic networks. For example, to model the metabolic reaction *A *+ *B *⇌ *C *+ *D *as an undirected hypergraph, we take *V *= {*A*, *B*, *C*, *D*}, and ℰ = {*E*}, where *E *= *V*. To distinguish between the two sets of metabolites on opposite sides of a reaction, a hyperedge *E *can be further bipartitioned into two subsets *E_t_*, the *tail set*, and *E_h_*, the *head set*. In this case, we write *E *as the ordered pair (*E_t_*, *E_h_*), and the direction of the edge is, by convention, from the tail set to the head set. Using this notation of directed hyperedges, a *directed hypergraph *is defined. For example, the hyperedge corresponding to the irreversible reaction *A *+ *B *→ *C *+ *D *is the ordered pair *E *= ({*A*, *B*}, {*C*, *D*}).

The *degree d*(*v*) of a node *v *∈ *V *in a hypergraph is defined as the cardinality of the set {*E *∈ ℰ | *v *∈ *E*}. The *neighborhood *of a node *v*, denoted by *N*(*v*), in a hypergraph is defined as the node *v *itself together with the set of all nodes connected to it by a hyperedge. More formally,(1)

The neighborhood of a set of nodes, *U*, is defined as the union of the neighborhoods of all nodes in *U*, or . Further, we denote by ℳ(*v*) the set of hyperedges of which *v *is an element, that is, ℳ(*v*) = {*E *∈ ℰ | *v *∈ *E*}.

### From Hypergraphs to Standard Graphs

A variety of transformations can be applied to a hypergraph to obtain standard graph representations. We now define transformations that are applicable (and have been applied) in the context of representing metabolic networks. Let *H *= (*V*, ℰ) be a hypergraph. The *primal *of *H *is a (standard) graph *G_p _*= (*V*, *E_p_*), where every two nodes in *V *that are connected by a hyperedge in *H *are connected by an edge in *G*. In other words,

The primal of a metabolic hypergraph is also called the *substance model *[12], since every pair of substances (metabolites) participating in the same reaction are connected by an edge (i.e., form a *clique*). For directed hypergraphs, primal graphs can be defined in two ways. The *cis*-primal is obtained by connecting with an edge every pair of nodes within the same partition of the hyperedge (both nodes from the head set or both from the tail set). In other words, the *cis*-primal of *H *is a graph *G_cp _*= (*V*, *E_cp_*), where(2)

This corresponds to the *substrate-substrate model *[12], where metabolites on the same side of a reaction are connected. The *trans*-primal is obtained by connecting with an edge every pair of nodes that belong to two different parts of a hyperedge (one from head set and the other from the tail set). In other words, the *trans*-primal of *H *is a graph *G_tp _*= (*V*, *E_tp_*), where(3)

This corresponds to the *substrate-product model *[12], where metabolites on opposite sides of a reaction are connected. Figure 1 illustrates these three transformations (See additional file 1 for an illustration on a real and small metabolic model, the catabolism of tagaturonate).

Every undirected hypergraph can be completely described by a binary matrix *M*, called the *incidence matrix*, where columns correspond to hyperedges and rows to nodes. An entry *M*[*i*, *j*] = 1 denotes that node *i *is an element of hyperedge *j *while an entry *M*[*i*, *j*] = 0 denotes otherwise (Notice that a stoichiometric matrix is a weighted incidence matrix of a metabolic network's connectivity map.). A binary matrix is a valid incidence matrix if and only if every row and column contains at least one 1. Thus, the transpose of the incidence matrix of any hypergraph is also a valid incidence matrix. The transpose of the incidence matrix of a hypergraph *H *corresponds to the *dual hypergraph H'*. The common practice of creating a reaction graph by connecting two reactions if they share a reactant [12] (also known as the *physical line graph transformation *[30], or PLGT for short hereafter) amounts to first computing the dual of the original metabolic hypergraph, and then taking the primal of the resulting hypergraph (see Figure 1).

Finally, common set operations, such as union and intersection, can also be introduced into the hypergraph transformation. One of the widely, yet implicitly, used case is the generation of enzyme/gene hypergraphs from the underlying reaction hypergraph [2]. Each hyperedge in the transformed hypergraph is the union of all hyperedges corresponding to reactions that are catalyzed by some particular enzymes/genes. This process is equivalent to resampling a number of subsets of the set of all hyperedges. Note that unlike reaction hyperedges, these hyperedges may substantially overlap or even coincide with each other (when multiple enzymes/genes catalyze a same set of reactions).

### Clustering Coefficients on Hypergraphs

A commonly used statistic for elucidating properties of metabolic networks, such as modularity [29] and small-worldness [32], is the *clustering coefficient*. Among the various existing definitions of the clustering coefficient, the *local clustering coefficient *by Watts and Strogatz [32] and the *global clustering coefficient *by Barrat and Weigt [33] are the most widely used.

According to [32], the local clustering coefficient, *C*_local_, for any given node *v *(with *d*(*v*) ≥ 1) in an undirected (standard) graph is defined as the fraction of the number of edges linking pairs of *v*'s neighbors over the number of all such possible edges (which equals ). For a node with *d*(*v*) = 0, we have *C*_local_(*v*) = 0. Intuitively, *C*_local _measures, for a node *v*, the probability that a randomly chosen pair of its neighbors would be seen connected.

According to [33], for an undirected graph with at least one 2-path (three distinct nodes connected via two edges), the global clustering coefficient *C*_global _is defined as the fraction of the number of 2-paths with linked end points (i.e., triangles) over the number of all possible 2-paths. Intuitively, *C*_global _measures the probability of having an edge (*u*, *w*), given that edges (*u*, *v*) and (*v*, *w*) exist, with *u*, *v*, *w *being three distinct nodes.

For a proper extension of *C*_local _and *C*_global _to the domain of hypergraphs (denoted by *HC*_local _and *HC*_global_, respectively), the following intuitive properties may be desirable, in addition to reflecting the extent of clustering in a hypergraph:

P1 The values of *HC*_local _and *HC*_global _fall in the range [0, 1].

P2 *HC*_local _and *HC*_global _should reduce to *C*_local _and *C*_global_, respectively, when every hyperedge connects exactly two nodes (i.e., the hypergraph is a standard graph).

P3 *HC*_local_(*v*) should reflect the extent of connectivity among neighbors of *v *due to hyperedges other than ones connecting *v *with those neighbors.

The rationale behind property P1 is to retain the probabilistic interpretation of the clustering coefficient statistic, as well as to enable comparing two different hypergraphs under the statistic. The rationale behind property P2 is to allow treating hypergraphs and standard graphs (which are a special case of hypergraphs) in a uniform manner. Property P3 reflects the fact that neighbors of a node can also be neighbors simply since all three belong to the same hyperedge--a case that should be treated carefully to reflect a proper notion of clustering.

**Figure 9:**
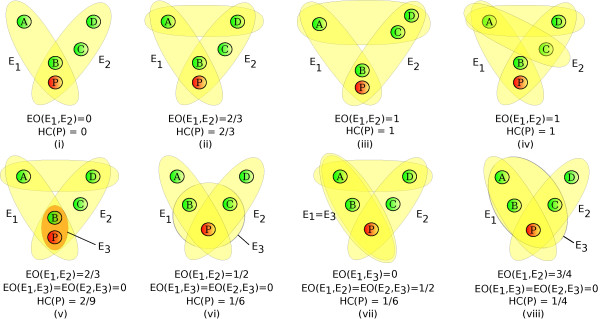
**Illustration of the *Extra Overlap *and the *Local Clustering Coefficient *for hypergraphs**. *EO*(*E_i_*, *E_j_*) denotes the extra overlap between hyperedge *E_i _*and *E_j_*, and HC(P) denotes the local hypergraph clustering coefficient for the node P.

Based on these properties, we define *HC*_local_(*v*) and *HC*_global_(*H*) as follows for a hypergraph *H *= (*V*, ℰ) and *v *∈ *V*:(4)(5)

where, ℐ = {{*E_i_*, *E_j_*} ⊂ ℰ | *E_i _*∩ *E_j _*≠ ∅ ⋀ *E_i _*≠ *E_j_*}, and the *extra overlap *of two intersecting hyperedges *E_i _*and *E_j _*is defined as:(6)

where *D_ij _*= *E_i _*- *E_j_*. For two hyperedges *E' *and *E" *such that *E' *= *E"*, we define *EO*(*E'*, *E"*) = 0. Figure 9 provides examples of the values of *EO *and *HC*_local _under a variety of scenarios. For *HC*_global_, the numerator is the sum of extra overlap between any pairs of hyperedges that contain *v*, and the denominator is the number of all possible pairs of such hyperedges.

From the definition of *EO*, we observe the following:

1. *EO*(*E'*, *E"*) ∈ 0[1] for every pair of hyperedges *E' *and *E"*.

2. For two non-identical, intersecting hyperedges, *E_i _*and *E_j_*, each of cardinality 2, *EO*(*E_i_*, *E_j_*) = 1 when their non-shared elements are linked by a third hyperedge, and *EO*(*E_i_*, *E_j_*) = 0 otherwise.

3. For any two sets *E*, *E' *⊆ *V*, where *E' *⊆ *E*, *EO*(*E*, *E'*) = 0.

It follows from these observations that *HC*_local _and *HC*_global _satisfy the three aforementioned properties P1--P3.

Note that we are not the first to define clustering coefficient measures for hypergraphs. Estrada and Rodríguez-Velázquez [24] defined their (global) clustering coefficient for hypergraphs, denoted ERV hereafter, as(7)

where a hyper-triangle is a set of three nodes and three hyperedges that connect them, and a 2-path is a sequence {*u*, *E*_1_, *v*, *E*_2_, *w*}, where *u*, *v*, *w *are three distinct nodes, *E*_1_, *E*_2 _are two distinct hyperedges, {*u*, *v*} ⊆ *E*_1 _and {*v*, *w*} ⊆ *E*_2_. The numerator is essentially the number of the closed-walks of length 3 without reusing hyperedges or revisiting nodes except at the end points [24].

**Figure 10:**
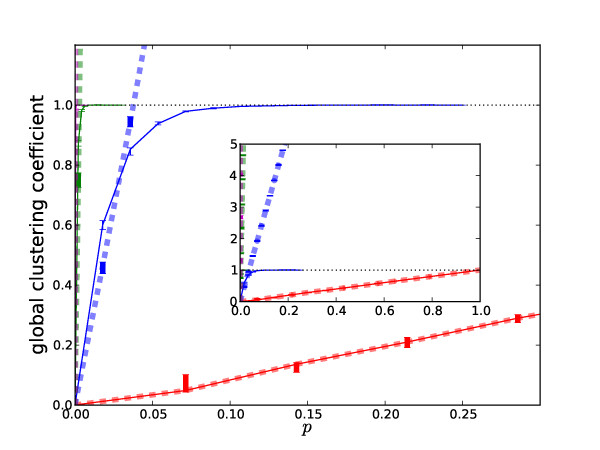
**Comparison of the two global hypergraph clustering coefficient measures on random hypergraphs**. The x-axis shows the probability *p *with which a hyperedge with a fixed cardinality is added, and the y-axis shows the value of the global clustering coefficient. Each hypergraph has 30 nodes. Solid and dashed lines correspond to our measure (Eq. 5) and the ERV measure (Eq. 7). Red, blue, green and magenta colors correspond to hyperedge cardinalities 2, 3, 4, and 5, respectively. A completely connected hypergraph has *p *= 1. Note that for |*E*| = 2 (red) the two coefficients agree and both degenerate into the standard graph clustering coefficient [33]. Each data point shows the median of 15 replica and the error bar shows the upper and lower quartiles.

To analyze how the two measures of global clustering coefficients compare, we conducted a simple test, where we generated random hypergraphs with increasing connectivity and applied the measures to them. More precisely, we generated a random graph by starting with 30 disconnected nodes, and then, for each subset of *m *nodes, we connected them by a hyperedge with probability *p*. Finally, we applied the two measures to the generated graph. In our experiment, we used *m *= 2, 3, 4, 5 and varied *p *between 0 and 1, and for each combination of values of *m *and *p*, we repeated the experiment 15 times, plotting the median of the 15 runs in Figure 10.

Three observations are in order. First, the two measures yield identical results in the case of standard graphs (where *m *= 2), since they both reduce to the standard global clustering coefficient statistic on standard graphs when all hyperedges have cardinality 2. Second, the two measures begin to deviate as our measure approaches 1. In particular, the ERV measure is not bounded from above (see additional file 1 for a discussion), and goes beyond 1 quickly for hyperedge cardinality higher than 2. This makes hard the interpretation of values computed by the ERV measure, since they cannot be treated in a probabilistic manner. Further, the ERV measure would not allow for comparing two hypergraphs in terms of their clustering coefficients since the values are not bounded. Last but not least, in both definitions of the hypergraph clustering coefficient, the hypergraphs with higher hyperedge cardinalities approach 1 much faster in their global clustering coefficient. The reason for this is that the total number hyperedges of a given hyperedge cardinality (which equals ) grows exponentially with the hyperedge cardinality value |*E*|. Therefore, the density  is diminished by the same factor if the number of hyperedges |ℰ| is kept fixed. This further illustrates the fact that hyperedge cardinality plays a significant role in the clustering coefficient computed on hypergraph and beyond. Similar patterns were observed for the local clustering coefficients measures (see additional file 1).

## Authors' contributions

All authors read and approved the final manuscript.

## Supplementary Material

Additional file 1 **Additional Information**. The file contains additional information on methods for null model generation, reaction size distribution for four more organisms, other abstraction methods as well as their illustration on a concrete metabolic pathway, discussion on currency metabolites and on other clustering coefficients defined on hypergraphs. Click here for file
