# Supplementary material for: Properties of metabolic graphs: biological organization or representation artifacts?
Source: BMC Bioinformatics. 2011 May 4;12:132. doi: 10.1186/1471-2105-12-132 (PMC3098788; doi:10.1186/1471-2105-12-132)
Supplement: Additional file 1 — Additional Information. The file contains additional information on methods for null model generation, reaction size distribution for four more organisms, other abstraction methods as well as their illustration on a concrete metabolic pathway, discussion on currency metabolites and on other clustering coefficients defined on hypergraphs. [file 1471-2105-12-132-S1.PDF]

Additional Information

W. Zhou and L. Nakhleh

1 Two methods of generating random hypergraphs of a fixed hyperedge cardinality.

Knowing the number of nodes n, the number of hyperedges m and the number of edges ℓ in the primal of the *E. coli* metabolic hypergraph, we generated random hypergraphs of fixed cardinality k in two different ways. One is to sample the same number of hyperedges as $\ell/\binom{k}{2}$. The method usually generates random hypergraphs with their primals having slightly lower numbers of edges due to the hyperedge overlap. To better keep the number of edges in the primal of the randomly generated hypergraph, we applied the second method which samples as many hyperedges as necessary on the fly so that the number of edges in the primal approximates the number of edges in the primal of the *E. coli* metabolic hypergraph up to k. A closer inspection of the results reveals the following relationship: When $k = 2$, both methods generate hypergraphs with primals having $m_1 = m_2 = 5719$ edges as expected. When $k = 3$, the second method gives m_2 fluctuates from 1911 to 1919 which is only slightly higher than $m_1 = 5719/\binom{3}{2} = 1906$ as in the first method. When $k = 4$, $m_2 = 956 \sim 960$ which is also slightly higher than $m_1 = 5719/\binom{4}{2} = 953$. When $k = 5$, $m_2 = 574 \sim 576$ which is also slightly higher than $m_1 = 5719/\binom{5}{2} = 571$. When $k = 6$, $m_2 = 383 \sim 384$ which is also slightly higher than $m_1 = 5719/\binom{6}{2} = 381$. The numbers of all possible hyperedges that can exist for hyperedge cardinality 3,4,5,6 are of the scale 10^8, 10^{10}, 10^{13} and 10^{15}. Therefore the hypergraphs with 1168 hyperedges are extremely sparse. Although the chance of hyperedge overlap is still very high, from what we observe from experiments the expected number of hyperedges that would produce ℓ (the number of edges produced from a hypergraph where there is no overlap) is only slightly higher than m (the number of hyperedges of such hypergraph). Given the specific combination of m, n and ℓ in *E. coli* metabolic hypergraph, the second method renders very similar results (Fig. 1) as by the first method (shown in the main text).

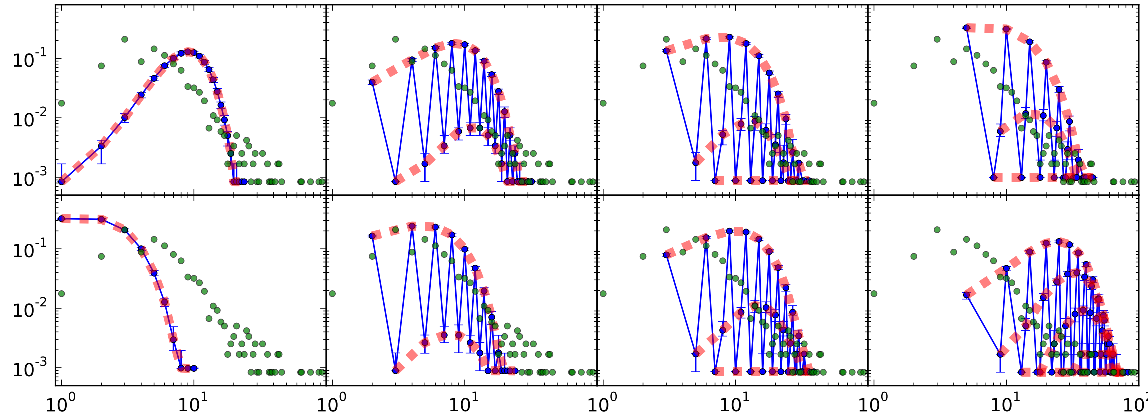

Figure 1: **The degree distributions of the primal graphs of random hypergraphs.** Each of the hypergraphs has 1193 nodes and 1168 hyperedges. Columns from left to right correspond to fixed hyperedge cardinalities of 2, 3, 4, and 5, respectively. The results in each panel are based on the 300 randomly generated hypergraphs (replica). For each well represented degree value (contained in at least 10 replica), the median is plotted. Error bars indicate quartiles. Green dots correspond to the degree distribution of the primal graph of the (undirected) metabolic hypergraph of *E. coli*. All plots are on log-log scales.

2 The hyperedge cardinality distribution of more organisms.

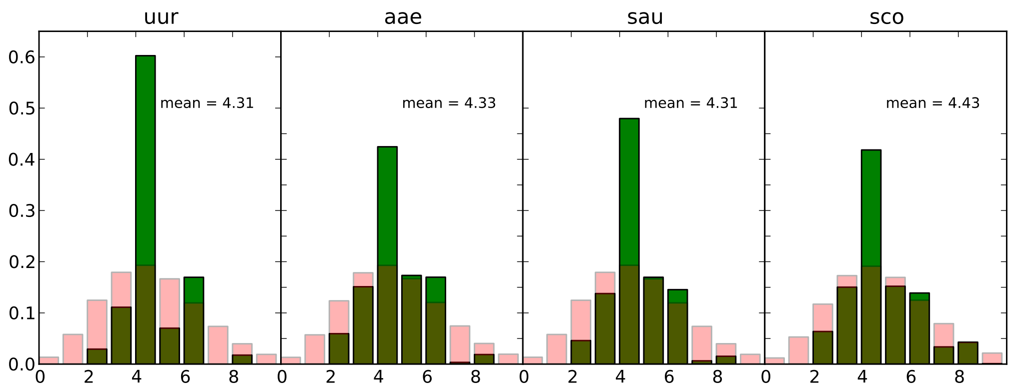

Figure 2: The hyperedge cardinality distribution of more species: *Aquifex aeolicus* (aae), *Staphylococcus aureus* N315 (sau), *Streptomyces coelicolor* (sco) and *Ureaplasma urealyticum* (uur).

3 The scaling of clustering coefficient of multiple abstraction methods

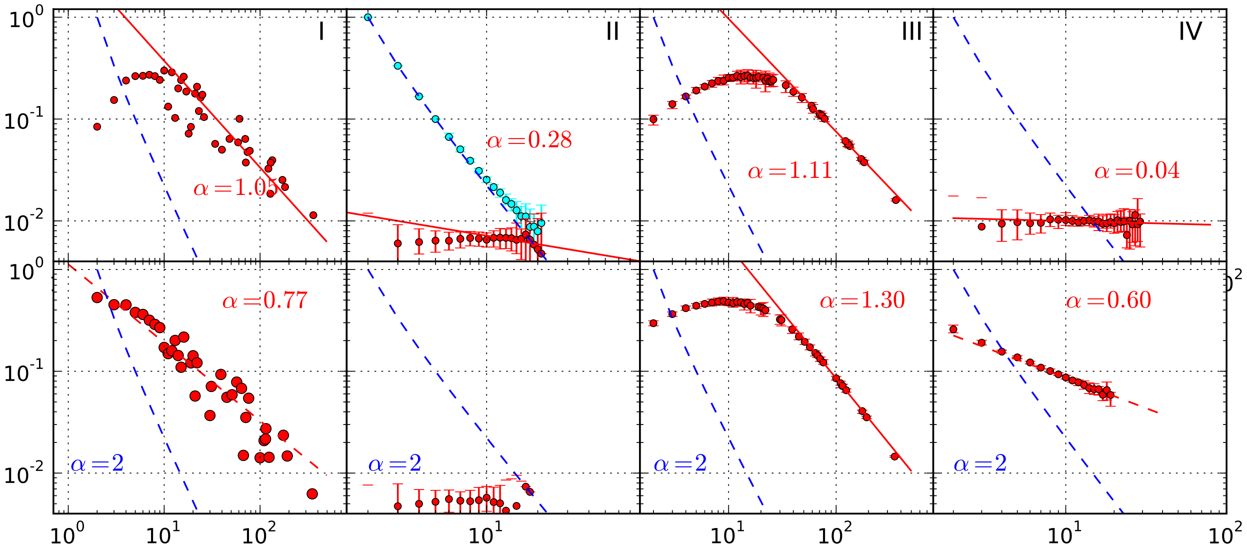

Figure 3: The scaling of clustering coefficient of mutliple abstraction methods. The upper row: *trans*-primal of directed hypergraph. The bottom row: *cis*-primal of directed hypergraph. Note that in the upper row the rewired hypergraph gives very different shape in $C(k)$. This is because the *trans*-primal no longer preserves a clique during the randomization. The bottom row shows similar result as the primal since the *cis*-primal preserves a clique structure.

4 Averaged clustering coefficients of dual metabolic hypergraph under continuous removal of "currency metabolites"

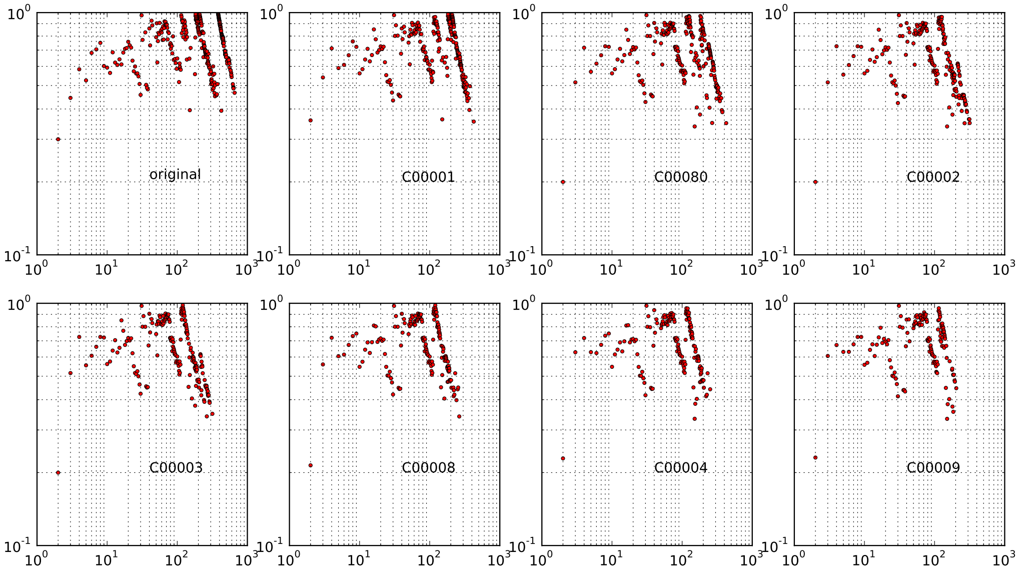

Figure 4: Averaged clustering coefficients under continuous removal of "currency metabolites", where the labels are KEGG compound indices. (Upper row, from left to right, corresponds to original, water, proton, ATP. Bottom row, from left to right corresponds to NAD+, ADP, NADH and phosphate).

5 $C(k)$ against k on the PLGT graph and its rewired counterparts.

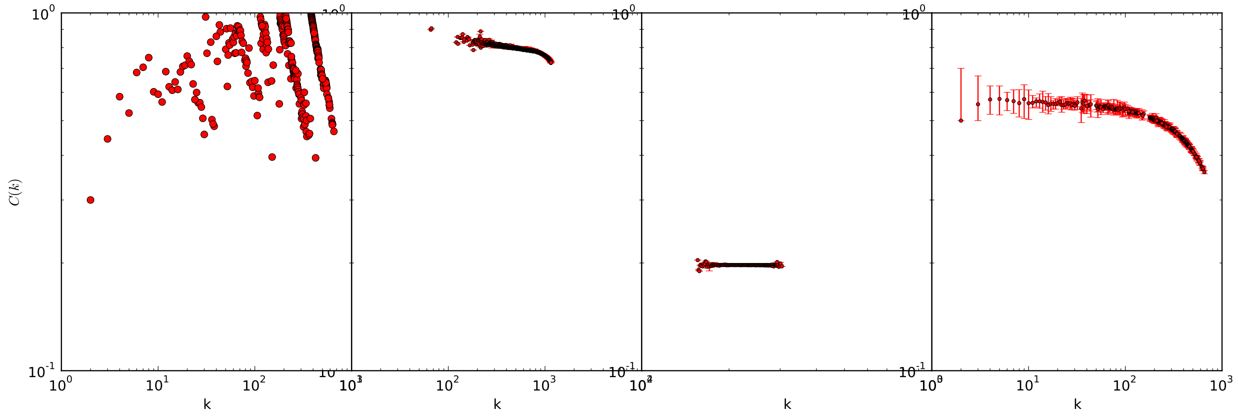

Figure 5: **The rewiring of PLGT graph of the primal of *E. coli* metabolic hypergraph.** From left to right: **1)** PLGT of the primal of the original *E. coli* metabolic hypergraph. **2)** The *E. coli* metabolic hypergraph was first rewired then undergoes PLGT. **3)** The rewired graph of 1) without keeping the degree distribution. (Erdös-Renyi graph of the same node and edge number) **4)** The rewired graph of 1) keeping the degree distribution. Each rewired graph undergoes 100000 rewiring operation. Convergence is checked (data not shown).

6 Hypergraph clustering coefficients computed using Estrada and Rodríguez-Velázquez (ERV) fashion

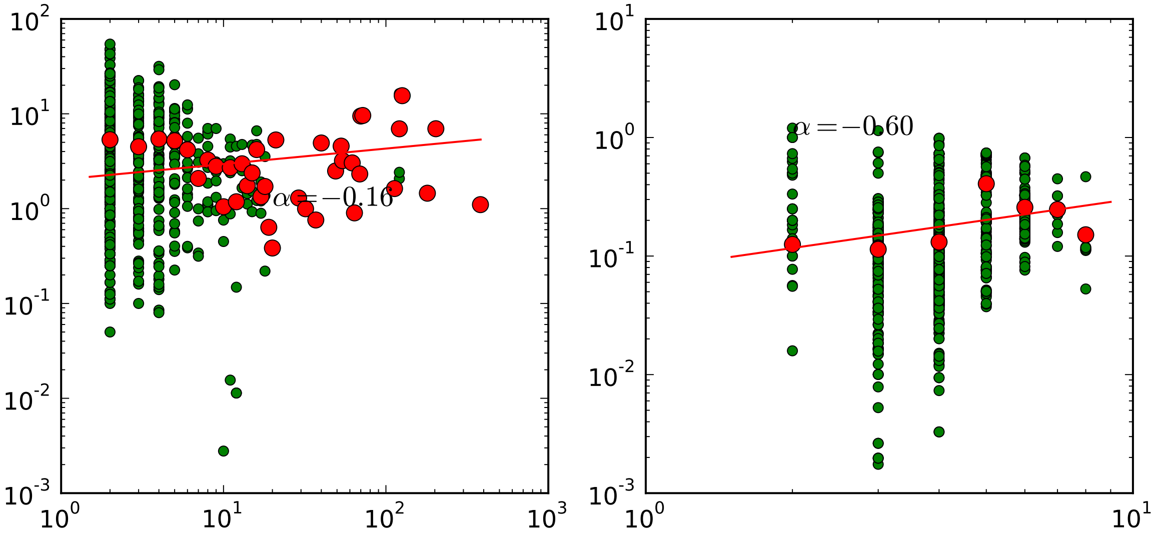

Figure 6: Hypergraph clustering coefficients of the *E. coli* metabolic hypergraph and its dual. Hypergraph clustering coefficients is computed in Estrada and Rodríguez-Velázquez fashion.

7 Illustration of hypergraph abstractions using a specific metabolic network (the catabolism of tagaturonate).

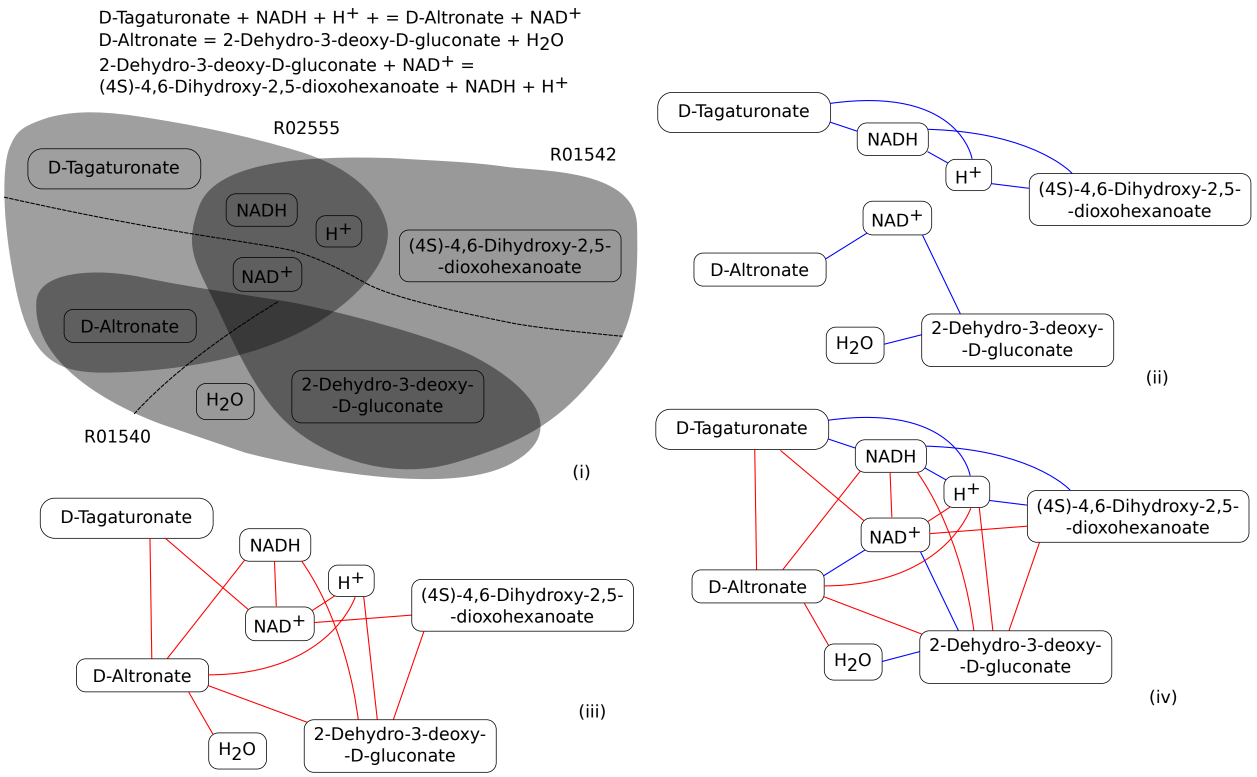

Figure 7: **Illustration of the abstraction of metabolic hypergraph using a specific pathway.** The model network is extracted from the catabolism of tagaturonate. It is composed of three reactions, namely R02555, R01540 and R01542 in KEGG's notation [KG00]. i) the hypergraph representation of the reaction system shown above. Three standard graph abstractions are in order: the *trans*-primal (ii), the *cis*-primal (iii) and the primal (iv).

8 Proof that the hypergraph clustering coefficient by Estrada and Rodríguez-Velázquez is unbounded as the hyperedge cardinality increases.

To see this, consider a hypergraph of n nodes and $\binom{n}{n-1} = n$ distinct hyperedges with cardinality $n-1$, we compute its Estrada's clustering coefficient using symmetry. First, the total number of 2-paths can be counted in the following formula:

$$2 \times n \times \binom{n-1}{2} \times N_p \tag{1}$$

where N_p is the number of 2-paths taking the form $\{a, E_i, p, E_j, b\}$, E_i, E_j are two hyperedges that include node p and connect it to node a and b respectively. $\binom{n-1}{2}$ is the total number of such choice of E_i and E_j. n is the total number of such choice of p. 2 takes into account of two directions of each path. By dividing E_i into $E_i \setminus E_j$ and $E_i \cap E_j$, N_p reads:

$$\begin{aligned} N_p &= |E_i \setminus E_j| \times (|E_j| - 1) + (|E_i \cap E_j| - 1) \times (|E_j| - 2) \\ &= 1 \times (n-2) + (n-3) \times (n-3) \\ &= n^2 - 5n + 7 \end{aligned}$$

Similarly, the total number of hyper-triangles can be counted using the following formula:

$$2 \times n \times \binom{n-1}{2} \times M_p \tag{2}$$

where M_p is the number of hypertriangles taking the form $\{a, E_i, p, E_j, b, E_k\}$. In addition to the process followed in counting 2-paths, E_k denotes the number of hyperedges that connect a and b but distinct from both E_i and E_j. By dividing the E_i into $E_i \setminus E_j$ and $E_i \cap E_j$, E_j into $E_j \setminus E_i$ and $E_j \cap E_i$, M_p reads,

$$\begin{aligned} M_p = \; & |E_i \setminus E_j| \times |E_j \setminus E_i| \times (n-2) \\ & + (|E_i \cap E_j| - 1) \times |E_j \setminus E_i| \times (n-3) \\ & + (|E_i \cap E_j| - 1) \times |E_i \setminus E_j| \times (n-3) \\ & + (|E_i \cap E_j| - 1) \times (|E_i \cap E_j| - 2) \times (n-4) \\ = \; & 1 \times 1 \times (n-2) + (n-3) \times 1 \times (n-3) \times 2 \\ & + (n-3) \times (n-4) \times (n-4) \\ = \; & n^3 - 9n^2 + 29n - 32 \end{aligned}$$

Putting the two parts together, the clustering coefficient by Estrada et al. on this hypergraph equals

$$\frac{M_p}{N_p} = \frac{n^3 - 9n^2 + 29n - 32}{n^2 - 5n + 7} \tag{3}$$

which is unbound above as n increases.

9 The global and local hypergraph clustering coefficient

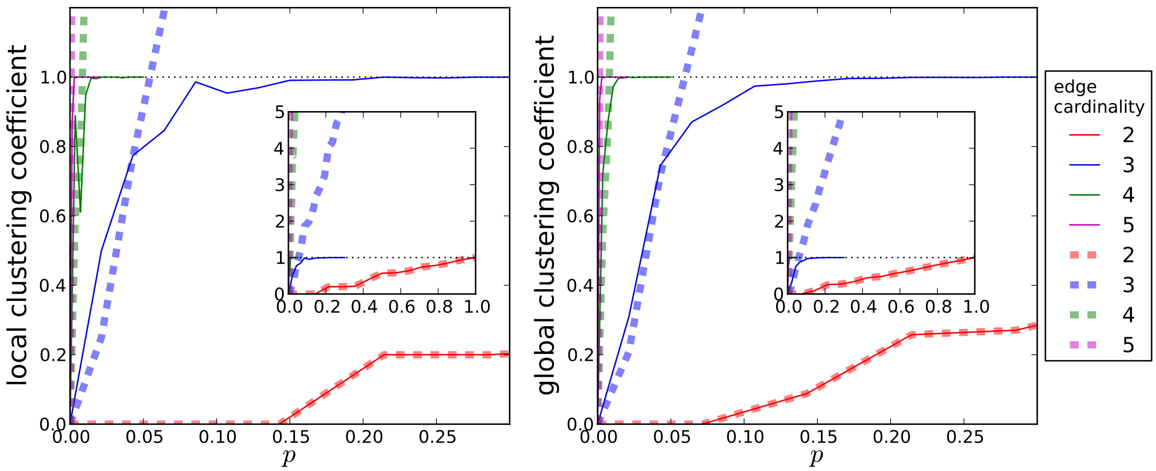

Figure 8: **Comparison of the two hypergraph clustering coefficient measures on random hypergraphs.** The x-axis shows the probability p with which a hyperedge with a fixed cardinality is added, and the y-axis shows the value of the local (**Left**) and global (**Right**) clustering coefficient. Each hypergraph has 30 nodes. Solid and dashed lines correspond to our measure and the ERV measure. Red, blue, green and magenta colors correspond to hyperedge cardinalities 2, 3, 4, and 5, respectively. A completely connected hypergraph has $p = 1$. Each data point shows the median of 15 replica and the error bar shows the upper and lower quartiles.

References

[KG00] Minoru Kanehisa and Susumu Goto. KEGG: kyoto encyclopedia of genes and genomes. *Nucl. Acids Res.*, 28(1):27–30, 2000.
